# Use of health services by adults in Manaus, 2019: Protocol of a population-based survey

**DOI:** 10.1097/MD.0000000000015769

**Published:** 2019-05-24

**Authors:** Marcus Tolentino Silva, Bruno Pereira Nunes, Tais Freire Galvao

**Affiliations:** a Faculty of Medicine, Federal University of Amazonas, Manaus; b Post-Graduate Program of Pharmaceutical Sciences, University of Sorocaba, Sorocaba; c Department of Nursing in Public Health, Federal University of Pelotas, Pelotas; d Faculty of Pharmaceutical Sciences, University of Campinas, Campinas, Brazil.

**Keywords:** cross-sectional, diagnosis of health situation, population, surveys and questionnaires

## Abstract

**Introduction:**

Health services utilization is an indirect measure of the rights and equity of a health system. A 2015 survey conducted in the Manaus metropolitan region showed that in the previous year, over 70% of adults visited the doctor and 1 in 3 had visited a dentist. Socioeconomic factors and inequality played a central role in the usage of healthcare services and health situation in this population. Since then, political and economic crisis are evolving in Brazil. This project aims to estimate the prevalence of use of health services and the health status of the adults residing in Manaus in 2019.

**Methods and analysis:**

This is a population-based survey of adults (≥18 years old) residing in Manaus. This survey will be conducted in the first half of 2019 with 2300 participants who will be interviewed at home, selected from a probabilistic sampling in 3 stages (census tracts, household, and dweller), and stratified by sex and age quotas based on official estimates. The participants will be interviewed using previously validated tools and questions employed in Brazilian official surveys, which will cover use of health services and supplies, health status, and lifestyle. Primary outcome will be any healthcare usage in the last 15 days. Associations between health services usage and socioeconomic data and health outcomes will be assessed using a Poisson regression with a complex sampling design correction. Results will be reported according to the strengthening the reporting of observational studies in epidemiology statement.

**Ethics and dissemination:**

This project was approved by the Ethics Committee of the Federal University of Amazonas, Manaus, Amazonas, Brazil. All participants will sign an informed consent before the interview. The results will be disseminated in peer-reviewed manuscripts, reports, conference presentations, and through the media.

## 1 Introduction

The use of health services is an indirect method of measuring the access and equity of the health system. It can be measured in a general way – use of any health service – or specifically, by the most common health services.^[1]^ Lower access to health services and reduced awareness of chronic diseases lead to a lower life expectancy and healthy life expectancy in the population.^[2]^ The local burden of diseases also presses the need for health services, particularly noncommunicable diseases, which require continued assistance for secondary prevention and control.^[3]^ The environment and social context influence the burden of diseases and its associated costs, and its effects fall on the health system. The organization of the health system influences the health status of a population, which, in turn, affects the use of health services.

Investigation of the health situation and the use of these services are paramount to identify aspects that require improvement. In areas where this situational diagnosis is poor, like the Amazon, such efforts represent research priorities. In these geographically and economically disadvantaged settings, the system performance may be more vulnerable to unfavorable context.

In 2015, a first local effort was undertaken in the Manaus metropolitan region to better understand the health situation of the area.^[4]^ The use of health services and supplies revealed that socioeconomic inequities played an important role on health services usage in the previous year,^[5]^ with lower rates than the Brazilian average.^[6]^ More than 14% of respondents reported pent-up demands of surgeries, mainly because of lack of availability in the public health system.^[7]^ Self-medication with antibiotics was reported by a 5th of the people who used these prescription drugs in fortnight previous to interview.^[8]^

Chronic diseases were self-reported by more than half of adults, and multimorbidity, which was more frequent in women and the elderly, was reported by 29% of adults.^[9]^ The indigenous displayed a higher prevalence of chronic diseases; moreover, the browns, which comprise the largest ethnic group of the region, had less private health insurance coverage.^[10]^ Depressive symptoms were present in 7% of respondents^[11]^ and generalized anxiety disorder symptoms, in 8% of the sample^[12]^; both were associated with poorer health status. The survey also estimated the utility score (quality of life) in the population, which was higher in men and the richest individuals; this score provides useful data for clinical and economic evaluations of health outcomes in Brazil.^[13]^ Other results are under analysis for scientific publication.

Political and economic crises occurred in Brazil during the period after the survey had taken place, and austerity policies are currently ongoing.^[14]^ Beginning in 2017, social expenditure was frozen for 20 years, which is an unprecedented measure that is forecasted to increase child mortality by 9% by 2030.^[15]^ Changes in the Brazilian National Primary Healthcare Policy allowed municipalities to reallocate the primary care budget to other health initiatives and to reduce the size and components of family health strategy teams.^[16]^ By the end of 2018, Cuban doctors withdrew from *Programa Mais Médicos* (More Doctors for Brazil),^[17]^ which is a program to place doctors in remote and historically underserved areas of Brazil. Along with these major structural changes in the health system, social protection, food security, and fighting poverty programs are being dismantled.^[18]^ Neoliberal policies on labor rights and social security deregulations threaten the dynamics of this emerging economy and reduce social justice in a country marked by contrasts and inequalities, which are remnants from a history of slavery and military dictatorship.

These societal changes are expected to affect health services utilization and the health status of the population. A new population-based survey in 2019 would be useful to assess the early effects of ongoing austerity measures at the population level. This protocol states the planned methods for a cross-sectional, population-based study that aims to estimate the prevalence of health services usage and the health situation of adults residing in Manaus.

## 2 Methods

### 2.1 Study design

This is a protocol for a cross-sectional, population-based study to be held in Manaus in the first semester of 2019. The sample design will allow the representativeness of the adult population.

### 2.2 Setting

Manaus is the capital of Amazonas, the largest Brazilian state by territory. In 2018, the population was estimated at 2,145,444 inhabitants, comprising 53% of the state population.^[19]^ The city ranked 8th for Gross Domestic Product in 2016^[19]^ and 850th on the Human Developing Index among 5570 Brazilian cities in 2010.^[20]^ Sanitary sewage is adequate for 62% of households and urbanization for 26%.^[19]^ This capital of rainforest has 24% of urban afforestation.^[19]^ Manaus includes over 93% of the state's physicians – the highest concentration in Brazil – with a density of 2.15 doctors per 1000 inhabitants in 2017.^[21]^

### 2.3 Recruitment and sample size

All participants will be selected by probabilistic sampling in 3 steps and stratified by sex and age quotas defined by the official census.^[22]^ In the first step, 250 census tracts will be randomly selected out of the 2461 urban census tracts in Manaus.^[22]^ In the second step, households will be systematically sampled. A number between 1 and 20 will be drawn to determine the first household to visit; after this, 1 out of every 20 households will be visited until reaching the planned number of interviews in each census tract, which was based on the population of the neighborhood. For closed households or those that refuse to participate, the house to the right will be approached, and if this house is also unavailable, the same procedure will be performed with the house to the left. In the third step, 1 dweller from each household will be randomly selected. In each selected household, all dwellers of 18 years old or more will be listed, and 1 dweller will be chosen and interviewed based on sex and age quotas obtained from official estimates.^[23]^

From the 2015 survey estimate of 20% of any healthcare usage in the last 15 days,^[4]^ a confidence level of 95%, an absolute precision of 2%, a design effect of 1.5, and 2,106,355 inhabitants aged ≥18 years living in Manaus^[23]^; the sample size was calculated as 2300 participants. Field sampling will continue until reaching this sample size. To minimize the risk of differences in participants’ characteristics and those who decline to participate in the survey, daily goals will be stipulated based on predefined sex and age group quotas.

### 2.4 Variables

Independent variables to be measured in this study were based on previously validated tools and/or questions used in official Brazilian surveys, covering lifestyle, use of health services and supplies, and health status. We will collect the variables sex (male, female), age (in years), race/skin color (white, black, yellow, brown [Brazilian mixed race], indigenous), marital status (married, stable union, separated, divorced, widower, single), schooling (no schooling, incomplete elementary school, complete elementary school, incomplete middle school, complete middle school, incomplete high school, complete high school, incomplete higher education, higher education degree, post-graduation), social class (A, B1, B2, C1, C2, D/E, according to the 2018 Brazilian economic classification criteria),^[24]^ work status (unemployed, housewife, student, retired, domestic worker, military/policy/fireman, private sector employee, public sector employee, employer, freelancer, other), and religion (catholic, evangelical, spiritist, Afro-Brazilian [candomblé or umbanda], Buddhist, other, none).

### 2.5 Outcomes

The primary outcome will be the prevalence of health service usage in the 15 days prior to the interview. Secondary outcomes include the prevalence of other utilizations of health services, health situation and self-reported chronic diseases, and health risks (Table 1). To assess the prevalence of dependent variables, dummy variables will be built from the responses.

**Table 1:**
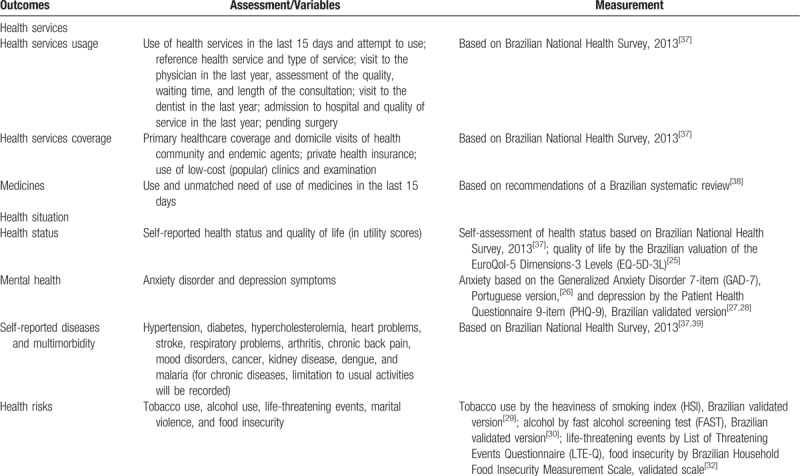
Outcomes, assessment, and measurement of the Manaus population-based survey, 2019.

Health service usage variables include use and attempt to use of health services in the last 15 days; use of the same health service (reference health service) and type of service; visit to the physician in the last year, assessment of the quality, waiting time, and length of the consultation; visit to the dentist in the last year; admission to hospital, and quality of service in the last year; primary healthcare coverage and domicile visits of health community and endemic agents; private health insurance coverage; use of low-cost (popular) clinics and diagnostics’ tests; use and unmatched need of use of medicines in the last 15 days; and pending surgery.

Health situation variables comprise self-reported health status (very good, good, fair, poor, very poor), quality of life (in utility scores),^[25]^ anxiety disorder,^[26]^ depressive symptoms,^[27,28]^ and self-reported diseases (hypertension, diabetes, hypercholesterolemia, heart problems, stroke, respiratory problems, arthritis, chronic back pain, mood disorders, cancer, kidney disease, dengue, and malaria). For chronic diseases, the degree of limitation to usual activities will be recorded to better assess morbidity and multimorbidity.

Health risks will consist tobacco use,^[29]^ alcohol use,^[30]^ life-threatening events,^[31]^ and food insecurity.^[32]^

**Figure 1:**
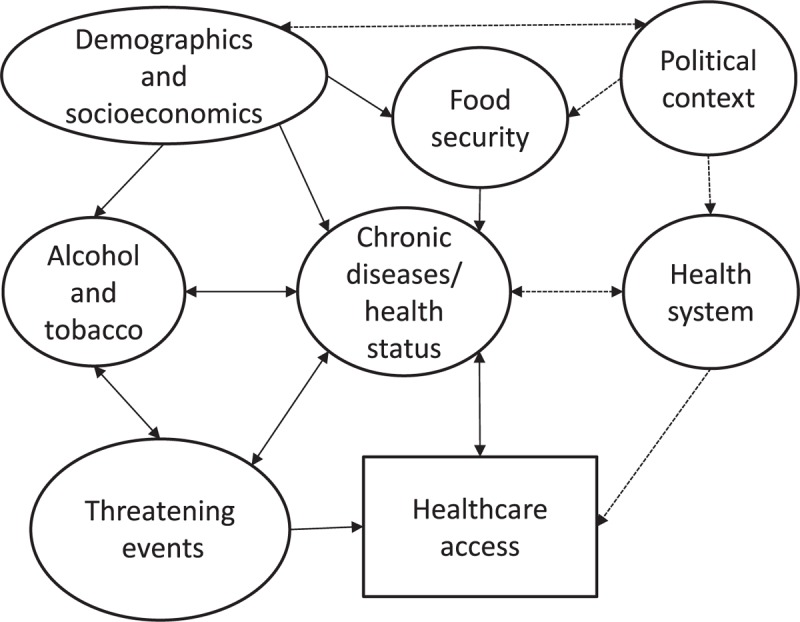
Hypothesized relationship of the outcomes to be assessed on the Manaus population-based survey, 2019.

A general relationship among outcomes is illustrated in Figure 1 to synthesize the intricate connection between the variables. The model contemplates contextual and individual variables related to healthcare access.

### 2.6 Measurements

Experienced interviewers will be contracted and trained by research team. All data will be recorded on questionnaires preconfigured on SurveyToGo software (Dooblo Ltd, Israel) using electronic devices (Intel TabPhone 710 Pro). The tablets will be synchronized over the internet to compile the database online on real time. A pilot survey will be held with 150 interviews to assess questionnaire comprehensiveness, which will compose the final sample. To ensure reliability, 20% of interviews will be audited by telephone. The interview will be georeferenced and partially recorded. As similarity of the options may desensitize the respondent, especially the latter questions, multiple response options will be ranked randomly at each interview to bring more consistency to responses.^[33]^

### 2.7 Data analysis plan

Descriptive statistics will be obtained for overall independent variables to describe the sample. The prevalence of the primary and secondary outcomes will be accompanied by 95% confidence intervals. When relevant, results will be stratified by sex and age. The data will be compared to the previous survey.

To identify the factors related to health services usage, a bivariate analysis will be conducted to calculate the prevalence ratio. Multicollinearity will be investigated by the tolerance indicator. To investigate the independent effect of the factors under investigation, a Poisson regression with robust variance will be adopted based on a hierarchical model including first distal and then proximal variables and compiled in blocks of analysis. Variables will remain in the model for the next step if a statistical significance of *P* < 0.1 is reached.

To investigate the association of secondary outcomes and independent variables, the nature of the dependent variable will be considered (continuous, ordinal, dichotomic, or multinomial),^[34]^ and a linear regression, Tobit regression, ordinal regression, or multinomial logistic regression will be performed on a hierarchical or multilevel model, taking variables of the neighborhoods and individuals into consideration to better explain contextual influence.^[35]^ All analyses will consider the complex sampling design.^[36]^ The statistical package Stata 14.2 (StataCorp, College Station, TX) will be used.

### 2.8 Ethics and dissemination

The project was submitted to the Ethics Committee of Federal University of Amazonas (Certificate of Presentation for Ethical Appreciation 04728918.0.0000.5020) and it was approved in Opinion no. 3.102.942 on December 28, 2018).

Theparticipants’ discomforts and risks include embarrassment in terms of identity by the interviewer, shame with personal questions, and a long interview (20–35 minutes). To prevent such situations, the surveyors will follow ethical recommendations, and confidentiality and comfort will be assured to respect the participants’ values and preferences.

The survey will begin after reading, comprehending, and signing the consent form. The participants’ names and other personal data that allow identification will not be published. The results will be presented in aggregate form, making it impossible to identify any of the participants. The survey maybe interrupted in case of major abnormality in the city like environmental disasters, tragedies, or catastrophe that thwarts the interviews. The researchers are experienced in population-based surveys.

Results will be disseminated on peer-reviewed manuscripts, conference presentations, and through the media. Unidentifiable data will be made available through in data repositories after each accepted manuscript.

### 2.9 Patient and public involvement

The contact with the public will be performed only in the recruitment of participants. The research questions and outcome measures were based in governmental surveys that were previously validated to Brazilian Portuguese. Results will be shared to the public by press releases to reach the lay media (newspapers, radio, and television).

## Author contributions

**Conceptualization:** Marcus Tolentino Silva, Tais Freire Galvao.

**Formal analysis:** Marcus Tolentino Silva, Bruno Pereira Nunes, Tais Freire Galvao.

**Funding acquisition:** Marcus Tolentino Silva.

**Methodology:** Marcus Tolentino Silva, Bruno Pereira Nunes, Tais Freire Galvao.

**Project administration:** Marcus Tolentino Silva, Tais Freire Galvao.

**Supervision:** Marcus Tolentino Silva, Tais Freire Galvao.

**Writing – original draft:** Marcus Tolentino Silva, Bruno Pereira Nunes, Tais Freire Galvao.

**Writing – review and editing:** Marcus Tolentino Silva, Bruno Pereira Nunes, Tais Freire Galvao.
